# Comparative study on seasonal variations in physico-chemical characteristics of four soda lakes of Ethiopia (Arenguade, Beseka, Chitu and Shala)

**DOI:** 10.1016/j.heliyon.2023.e16308

**Published:** 2023-05-18

**Authors:** Hana Melese, Habte Jebessa Debella

**Affiliations:** Department of Zoological Sciences, Addis Ababa University, Addis Ababa, Ethiopia

**Keywords:** Soda lakes, Physico-chemical parameters, Alkaline, Dilution, Evaporation

## Abstract

Soda lakes are productive natural ecosystems with substantial economic and non-economic values. Currently, they are facing considerable environmental threats that can lead to further degradation. The objective of this study was to investigate comparative spatiotemporal variations of physicochemical properties of four Ethiopian soda lakes in comparison with their historical data. Central (open-water) sampling sites were selected from four Ethiopian soda lakes: Arenguade, Beseka, Chittu and Shala. Water samples were collected from open sampling stations from January to December 2020 and analyzed at Limnology laboratory of Addis Ababa University. The geographical position of each lake was determined by means of Global Positioning System (GPS). All physicochemical factors exhibited significant differences between seasons (ANOVA, P < 0.05), except salinity in Lake Shala. The concentrations of physicochemical parameters were generally high during the dry seasons in the studied lakes due to the low incidence of rainfall, caused by recurrent drought, resulting in higher evapotranspiration rates as they are characterized by a long dry season. Lakes Arenguade and Beseka showed considerable decrease in conductivity, alkalinity and salinity, compared to data from the 1960s and 1990s, which might be attributed to dilution effect. The same parameters show slightly increasing trend in Lake Arenguade which might be due to high evaporation rate. In general, the physicochemical parameters of the study lakes showed temporal variations, which could be attributed to the dilution effect, evaporation, and hydrological characteristics of the Ethiopian Rift Valley. In the face of climate change and recurring droughts, in the Ethiopian Rift Valley, the outcomes of this study might be used as input for the long-term planning, of water resources management and devising mitigation strategies.

## 1 Introduction

Soda lakes are saline and alkaline ecosystems that are thought to have existed since the world's first geological records. They are extreme environments found all over the world, mostly, in arid and semi-arid regions. They are more common in the Eastern Rift Valley, which stretches from the Red Sea to Tanzania via Ethiopia and Kenya [1,2]. They are strongly alkaline aquatic environments with high conductivity (6000–160,000 μS/cm), extremely high pH (above 9.5), high salinity (>50g l^−1^) and high alkalinity due to sodium carbonate salt [[3], [4], [5]].

Since soda lakes are relatively closed systems, they are excellent environmental sensors, sensitive to weather and climate change. Even minor changes have a significant and, in some cases, irreversible impact on the natural character of these lakes [5]. Their physical and chemical properties are influenced by local geomorphology, basin characteristics, geochemistry and evaporation [2]. Monitoring water quality of soda lakes has become a high priority for the determination of current conditions and long-term trends for effective water management [6].

Ethiopia is rich in soda lakes, which are well-known for their unique diversity, scientific benefits, economic significance and scenic attractions. Presently, these lakes are under threat from both anthropogenic activities and natural events [7]. A change in the ecology of the catchment, such as water course diversion, sand mining, and water abstraction are adversely impacting the lakes’ ecosystem. On the other hand, climate change is affecting the physicochemical characteristics and biological community structure of the lakes, further complicating their ecology [8,9].

For instance, the water level of Lake Abijata is dropping due to soda ash extraction and upstream diversion for irrigation purposes, resulting in high salinities, change in biotic structure, and collapse in fish stock [[10], [11], [12]]. Furthermore, there is a plan to expand the Abijata soda ash manufacturing plant to Lake Shala. This will have a negative impact on the Limnology of Lake Shala [13]. In Lake Arenguade, dilution in lake chemistry, particularly salinity, conductivity, and alkalinity, have resulted in a dramatic decline in phytoplankton biomass and productivity. Underwater explosions for seismic experiments are considered to be major factor in the lake's general limnological change [14,15]. Lake Beseka has grown tenfold increase in size over the last 30 years, indicating a consistent change in the lake's chemistry with a decrease in total ionic concentration and electrical conductivity [16,17]. The primary cause of the lake's water level expansion was hypothesized to be discharge from hot springs [18], climate variability [19], groundwater input [20] and irrigation return water from adjacent farms [21].

The physicochemical properties of saline lakes naturally fluctuate due to seasonal changes in wet and dry seasons. These changes have direct impacts on the biota that live in saline lakes [22]. Increasing anthropogenic activities, climate change, and the dynamic nature of physicochemical parameters all necessitate the availability of contemporary limnological data. A number of authors have studied the physical and chemical characteristics of some Ethiopian soda lakes [13,14,16,[23], [24], [25], [26]]. Despite such studies at different times on separate lakes in the past, there are no such coherent studies that show trends in the limnological changes of the Ethiopian Rift Valley soda lakes in recent years. The objective of this study was to compare spatiotemporal variations of the physicochemical properties of four Ethiopian soda lakes in comparison with their historical data and identify trends of change. The results generated by this study will contribute to a better understanding of changes in the lakes’ ecosystem to devise effective management strategy and mitigation measures.

## 2 Materials and methods

### 2.1 Description of the study area

**Fig. 1:**
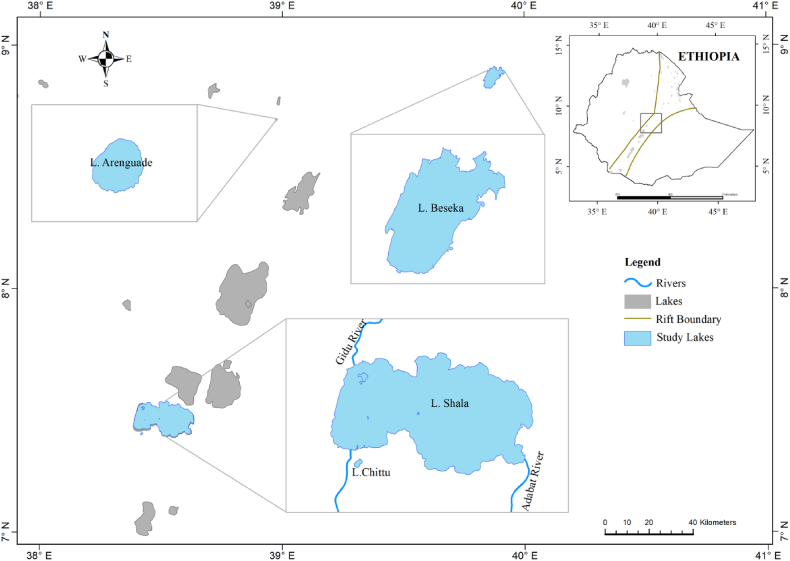
Map showing the study lakes.

This research was conducted in four Ethiopian soda lakes: Arenguade, Beseka, Chitu, and Shala (Fig. 1). Lakes Chitu, Beseka (Metahara), and Shalla are located within the Main Ethiopian Rift of the East African Rift Valley system, while Lake Arenguade is a crater lake on the Rift's western escarpment [15]. Monthly rainfall data for the sampling year (2020) was taken from Ethiopian National Meteorological Agency (ENMA). The morphometric characteristics of the study lakes were described in Table 1.

**Table 1:** Morphometric characteristics of the study lakes.

Parameter	Arenguade	Beseka	Chittu	Shalla
Latitude (N)	8^o^41.856′	8°51.5′	7^o^24′	7°28′
Longitude (E)	38^o^58.796′	39°51.5′	38^o^25′	38°30′
Altitude (m a. s. l)	1900	952	1600	1560
Area (km^2^)	0.54	40	0.8	370
Max. depth (m)	32	11.5	21	266
Mean depth (m)	18.5	6	-	86
Volume (km^3^)	0.010	23.31	-	37,000
Catchment area (km^2^)	-	401.5 (505)	-	3920
Basin origin	Volcanic crater	Volcanic crater	Volcanic crater	Tectonic

**Data source:** [2,25,[27], [28], [29]].

**Fig. 2:**
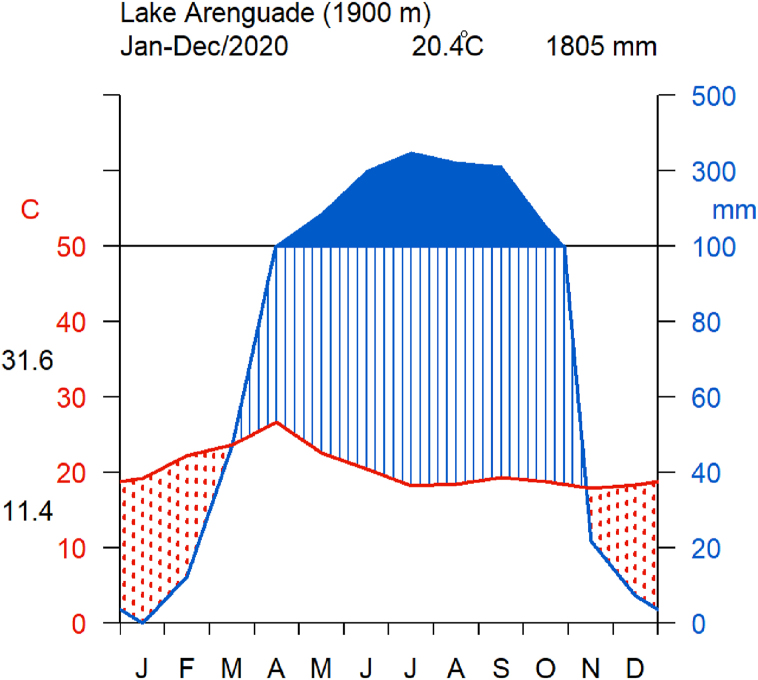
Walter and Lieth climate diagram showing a summary of climate conditions around Lake Arenguade during the year 2020.

Lake Arenguade (also known as Haro Hadho) is situated 45 km south of Addis Ababa. It is a small soda lake which is part of a large group of volcanic, explosion crater lakes known as the Bishoftu crater lakes [2]. The lake has a closed basin with no obvious inlet or outlet. Its crater rim rises about 200 m above the lake's surface. The average annual temperature is 20.4 °C; with a mean maximum of 31.6 °C and a mean minimum of 11.4 °C (EWNHS) (Fig. 2). The lake region experiences two rainy seasons: minor (February to April) and major (June to September). Lake Arenguade region period is from November to January [30]. It receives water primarily from rainfall that falls directly on its surface and run-off from its small catchments [14]. The mean annual rainfall of this area is 1805 mm (ENMSA). Lake Arenguade is named (Arenguade means green in Amharic language and Haro Hadho meaning salt lake in Afaan Oromo) for the dense production of the filamentous blue-green alga (*Arthrospira fusiformis*) [25].

**Fig. 3:**
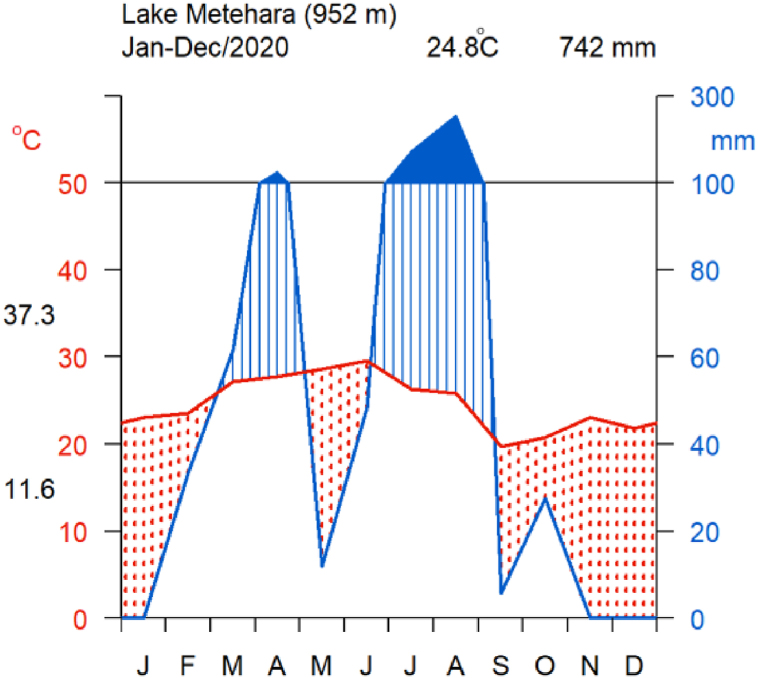
Walter and Lieth climate diagram showing a summary of climate conditions around Lake Beseka (Metehara) during the year 2020.

Lake Beseka (Metahara) is a volcanically dammed, shallow endorheic lake located about 200 km southeast of Addis Ababa [31]. The general climate of the lake's area is semi-arid with a mean annual temperature of 25 °C and a total mean annual rainfall of 534 mm [18]. The average annual temperature is 24.8 °C; with a mean maximum of 37.3 °C and a mean minimum of 11.6 °C (EWNHS) (Fig. 3). There are two main seasons: the rainy season (June–August) and the dry season (9 months) [32]. The mean annual rainfall of this area is 742 mm (ENMSA).

As the Lake is situated in the central rift valley region, it is vulnerable to the occurrences of different tectonic & volcanic activities [33].

**Fig. 4:**
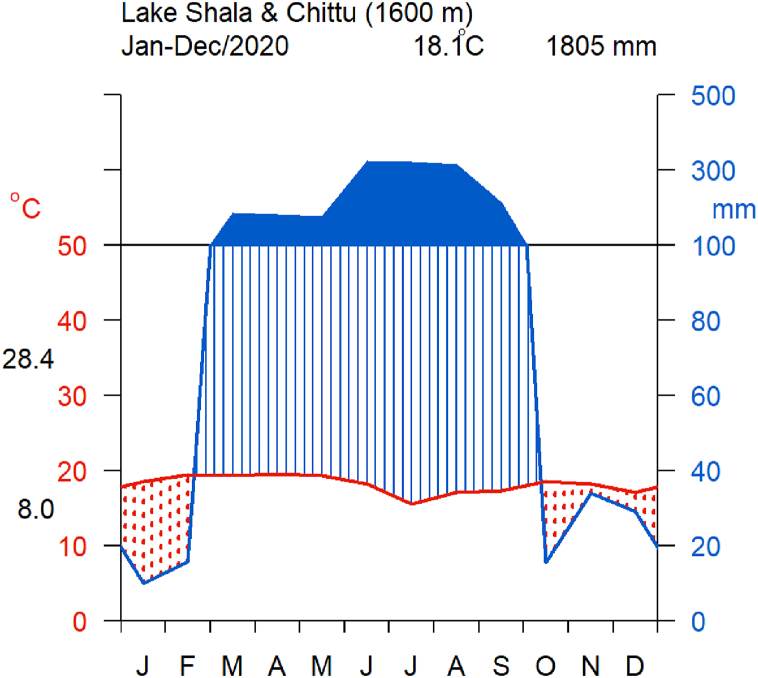
Walter and Lieth climate diagram showing a summary of climate conditions around Lake Shala and Chittu during the year 2020.

Lakes Chitu and Shala are located some 287 km south of Addis Ababa, within the Abijata-Shala Lakes National Park [30]. They are found in the hydrologically closed system of the Ziway-Shala basin, with no obvious surface outflows or inflows. Lake Chittu is fed by direct precipitation and a few hot springs on its shores [10] and Shala, is fed by direct precipitation, several hot springs and two rivers (Adabat and Gidu) [34]. The lakes’ region has semi-arid to sub-humid type of climate with mean annual precipitation and temperature of 600 mm and 25 °C, respectively [35]. The average annual temperature is 18.1 °C; with a mean maximum of 28.4 °C and a mean minimum of 8.0 °C (EWNHS) (Fig. 4). The region has two seasons: dry (October to February) and wet (March to September). The wet season is characterized by a bimodal pattern of rainfall, with minor rainy period extending from March to May and major rainy period from June to September. The mean annual rainfall of this area is 1805 mm (ENMSA). The region has higher rate of evaporation than precipitation, which causes rainfall deficit [36].

### 2.2 Sampling and analytical methods

#### 2.2.1 Physicochemical parameters

Water samples from four Ethiopian soda lakes: Arenguade, Beseka, Chitu and Shala, were taken from offshore sites (open water or center of the lake) and their geographical position was determined by means of Global Positioning System (GPS). Triplicate samples were collected from the study lakes surrounding each sampling site from 50 to 100 m distances from January to December 2020, four times in different seasons (Dry-January, Pre-rainy-June, Rainy-July, Post rainy-November). Over the whole sampling period, a total of forty eight samples (twelve from each lake) were gathered at midday.

Dissolved oxygen (DO), electrical conductivity (EC), pH and temperature were measured *in-situ* and vertical profiles were determined using a multi-metric probe (HQ40d). Prior to the analysis, the equipment was calibrated at Addis Ababa University's Limnology Laboratory using ionic strength corrected buffers to minimize the effect of ionic strength on pH and DO measurements. The conductivity measured *in-situ* was corrected to conductivity at 25 °C using a temperature coefficient of 2.3% per ^o^C [37]. Depth profiles were determined for three lakes considering their size and depth from 0, 1, 2, 3, 4, 5 m depths (Beseka and Chittu) and from 0, 1, 2, 3, 4, 5, 10, 15, 20 m depths (Shala).

Secchi depth (Z_SD_) was measured using a standard black and white Secchi disc of 20 cm diameter. The depth of the euphotic zone (Z_eu_) was calculated in meter from Z_SD_ reading using mathematical relationship: Z_eu_≈ 2.7*Z_SD_ [38]. Total suspended solids were estimated by weighing Whatman® Glass Microfiber Filters (GF/F), that had been pre-dried at 105 °C and calculated using the formula in literature [39]. Total alkalinity (TA) and phenolphthalein alkalinity (PA) were determined by titration of 100 ml of the sample with 0.1 N HCl to the appropriate end point pH using phenolphthalein (PA) and mixed bromocresol green and methyl red indicator for TA [40]. The levels of dominant ions (HCO_3_^−^ and CO_3_^2−^) were calculated using alkalinity values [41].

Water samples were collected using acid-washed 2 l polyethylene bottles. They were then transported to the laboratory in dark boxes, containing lake water that maintained temperature close to that in situ. Samples were filtered through GF/F and stored in polyethylene bottles near 4 °C for nutrient analyses, which were done within 48–72 h of sample collection. Nutrients were analyzed at Limnology Laboratory of Addis Ababa University following the standard methods [39]. All samples were determined manually by non-automated methods. Ammonia nitrogen (NH_3_–N) was determined by phenate method; soluble reactive phosphorus (SRP) and total phosphorus (TP) were determined using ascorbic acid method, after digesting the unfiltered sample using potassium persulfate. Nitrate (NO_3_–N) was determined using sodium-salicylate method and silica (SiO_2_) was determined by the Molybdo-silicate method. Total and phenolphthalein alkalinity of the lakes was determined by titration of the sample with 1 N HCl to pH = 4.5, using phenolphthalein and bromocresol green indicator solutions [40].

### 2.3 Data analysis

Statistical analysis was carried out using the R software (version 4.0.5) [42]. Significant differences among the results were determined using Kruskal–Wallis method of variance (ANOVA). The data collected were tested for normality using Shapiro-Wilk normality test and transformed to Log10 (X+1), where necessary, and differences were considered as significant when p < 0.05. Temporal variations in physicochemical parameters were described using bar plots (Sigma plot version 15). Walter and Lieth climate diagrams and line plots for depth profiles were constructed using ggplot2 (climatol and tidypaleo) functions respectively [43].

## 3 Results

### 3.1 Spatial variation of physicochemical parameters

The spatial variation of physicochemical parameters of the four study lakes have been tabulated (Table 2). The highest (25.9 °C) average water temperature recorded was at Lake Beseka, followed by Lakes Shala and Chittu, and the lowest temperature was recorded at Lake Arenguade. The average pH values recorded were high and showed little variation within the three lakes, whereas a low pH value was recorded in Lake Beseka. The highest measured dissolved oxygen (DO) was (7.36 + 0.72 mg l^−1^) measured in Lake Shala, followed by lakes Beseka, and Chittu but very low dissolved oxygen (3.33 + 0.24 mg l^−1^) was recorded in Lake Arenguade. Lake Arenguade had the highest turbidity, followed by Beseka and Chittu. Lake Shala, on the other hand, had the lowest turbidity. Secchi disk depth reached a maximum of 1.23 m in Lake Shala and a low of 0.3 m in Lake Arenguade. Lake Chittu had the highest conductivity, alkalinity, and salinity values, followed by Shala. In contrast, Lake Arenguade had the lowest values, followed by Lake Beseka (Table 2). In terms of nutrient concentrations in the lakes, ammonium nitrogen had the highest value (161.93 + 57.8 μg l^−1^) in Lake Arenguade and the lowest (56.39 + 14.69 μg l^−1^) in Lake Beseka. The minimum and maximum concentrations of nitrate nitrogen were 27.12 + 7.50 μg l^−1^ (Lake Shala) and 98.36 + 52.03 μg l^−1^ (Lake Beseka), respectively. Lake Chittu had the highest soluble reactive phosphate (SRP) 1.75 + 0.34 mgl^−1^ values, followed by Lake Shala and Arenguade. SRP concentrations in Lake Beseka were found to be low (0.44 + 0.16 mgl^−1^). Lake Chittu had the highest total phosphorus (TP) concentration (2.41 + 0.52 mg l^−1^), followed by Lake Arenguade and Shala, while Lake Beseka had the lowest. The lowest average 27.1 ± 1.72 meql^−1^ (HCO_3_^−^) and 29.53 ± 6.85 meql^−1^ (CO_3_^2−^) was recorded in Lake Beseka followed by Lake Arenguade and Shala. However, the highest average 313.1 ± 71.4 meql^−1^ (HCO_3_^−^) and 279.9 ± 27.4 meql^−1^ (CO_3_^2−^) was recorded in Lake Chittu.

**Table 2:** Limnological characteristics of the four Ethiopian soda lakes (mean ± sd) and range (min-max) during the present study period.

Parameters	Lakes			
	Arenguade	Beseka	Chittu	Shala
Temp (^o^C)	22.2 ± 0.55	25.9 ± 0.89	22.8 ± 0.58	22.9 ± 0.6
	21.5–23	24.7–27.5	22.2–23.5	21.9–23.4
pH	10.04 ± 0.34	8.55 ± 0.70	10.46 ± 0.14	10.12 ± 0.37
	9.57–10.41	7.87–9.31	10.2–10.6	9.8–10.7
EC (mS/cm)	5.42 ± 0.167	2.72 ± 0.123	66.9 ± 3.45	26.5 ± 2.94
	5.25–5.65	2.52–2.8	63.5–70.24	23–30.85
DO (mg l^−1^)	3.33 ± 0.24	6.61 ± 0.62	5.44 ± 0.27	7.36 ± 0.72
	3.12–3.66	6.01–7.21	5.14–6	6.63–8.47
Alk (meql^−1^)	50.2 ± 9.22	36.2 ± 3.45	426.7 ± 45.4	305.7 ± 23.27
	42–63.6	32–41.1	375.4–480	282–340
Sal (g l^−1^)	2.91 ± 0.50	1.88 ± 0.48	38.4 ± 7.60	16.78 ± 1.90
	2.1–3.3	1.42–2.38	29.7–45.6	14.4–19.5
Turbidity (NTU)	37.7 ± 4.00	35.9 ± 7.77	30.3 ± 7.66	15.05 ± 4.44
	33.5–42.6	25–44.5	22–38.7	10.8–22.14
Sechi depth (m)	0.3 ± 0.07	0.46 ± 0.05	0.49 ± 0.08	1.23 ± 0.04
	0.23–0.4	0.39–0.53	0.42–0.62	1.2–1.3
NO_3_–N (μg l^−1^)	40.6 ± 27.31	98.4 ± 52.03	69.5 ± 29.72	27.12 ± 7.50
	9.14–97.71	27.3–164.4	24.86–112	20.6–40.57
NH_3_–N (μg l^−1^)	161.9 ± 57.8	56.4 ± 14.69	113 ± 61.58	89.2 ± 39.2
	83–224.4	44.86–83.4	54.4–201.6	50.14–148.7
SiO_2_ (mg l^−1^)	39.4 ± 11.9	58.4 ± 37.10	2.59 ± 1.01	2.37 ± 0.25
	19.6–49.57	22.9–96.17	1.4–4.21	2.1–2.87
SRP (mg l^−1^)	1.04 ± 0.27	0.44 ± 0.16	1.75 ± 0.34	1.05 ± 0.50
	0.74–1.47	0.25–0.71	1.37–2.37	0.65–1.9
TP (mg l^−1^)	2.08 ± 0.51	1.15 ± 0.33	2.41 ± 0.52	1.59 ± 0.49
	1.48–2.8	0.75–1.49	1.79–3.21	1.1–2.3
HCO_3_^−^ (meql^−1^)	51.34 ± 9.17	27.1 ± 1.72	313.1 ± 71.4	140.73 ± 29.8
	41.3–63	22.8–29.95	224.02–389.6	102.3–170.5
CO_3_^2−^ (meql^−1^)	67.6 ± 17.6	29.53 ± 6.85	279.9 ± 27.4	141.82 ± 32.1
	41.6–79.14	24.35–38.96	239.45–299.5	102.27–175.3

### 3.2 Temporal variation of physicochemical parameters

**Fig. 5:**
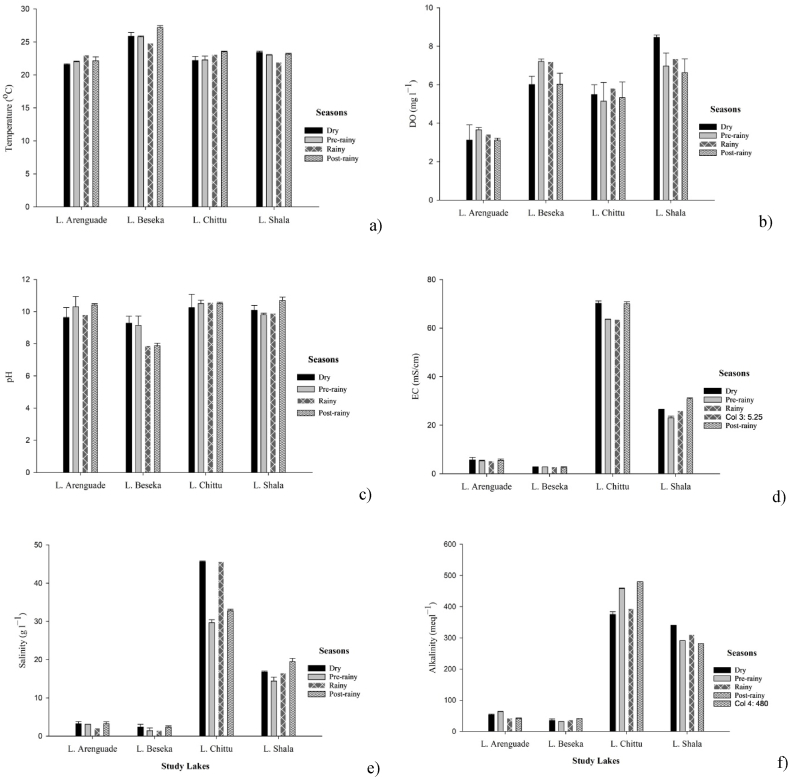
Seasonal variations in a) water temperature, b) dissolved oxygen, c) pH, d) conductivity, e) salinity and f) alkalinity of the study lakes during the study period.

**Fig. 6:**
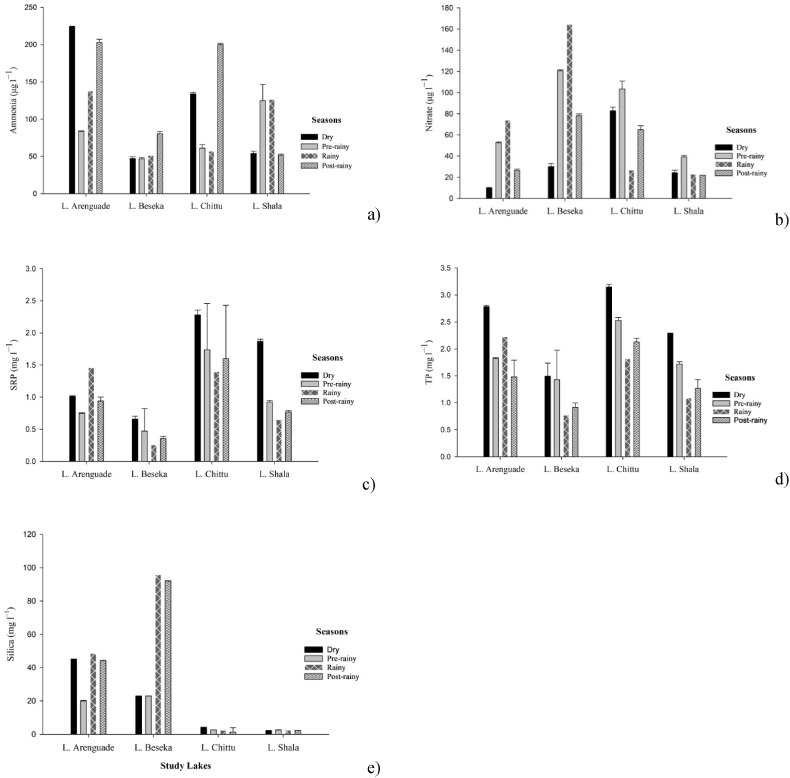
Seasonal nutrient variation in a) ammonia, b) nitrate, c) soluble reactive phosphorus, d) total phosphorus and e) silica of the study lakes during the study period.

The mean values of seasonal physico-chemical parameters recorded among the lakes during the study period are summarized in Fig. 5, Fig. 6. With the exception of variation in salinity of Lake Shala, all physicochemical variables showed significant differences between the sampling seasons (p < 0.05).

Temporal variations in temperature and dissolved oxygen concentration in Lake Arenguade indicated some variability among seasons. Temperature was high in post rainy seasons (23.0 °C) and low (21.57 °C) in pre rainy seasons while, dissolved oxygen concentration was high (3.66 mg l^−1^) in pre rainy seasons. Both electrical conductivity and pH values showed significant differences between sampling seasons. Electrical conductivity was high (5.65 mScm^−1^) in dry and low (5.25 mScm^−1^) in rainy season, whereas pH was high in post rainy season and low in dry season. Salinity results showed high values (3.24 g^−1^) during the dry season and low (2.10 g l^−1^) during the rainy season, while alkalinity results showed high values in the pre rainy season and low values in the post rainy season with the mean results of 63.3 meql^−1^,42.0 meql^−1^, respectively, showing significant variations between seasons (Fig. 5).

In terms of nutrient concentration in Lake Arenguade, nitrate and total phosphorus showed high values (73.9 μg l^−1^, 2.78 mg l^−1^) during the dry season, respectively. SRP concentrations were high (1.46 mg l^−1^) during the rainy season and low (0.75 mg l^−1^) during pre-rainy season. Ammonia concentrations show high values (224.0 μg l^−1^) during the post-rainy season and low values (84.4 μg l^−1^) during the pre-rainy season, and silica concentrations show high values (48.5 mg l^−1^) during the rainy season and low (20.0 mg l^−1^) during the pre-rainy season (Fig. 6).

In Lake Beseka, physicochemical parameters also showed significant differences between seasons (p < 0.05). pH values were high (9.28) during the dry season and low (7.87) during the rainy season. Electrical conductivity was higher (2.79 mS/cm) in the pre-rainy season and lower (2.52 mS/cm) in the rainy season. During the post-rainy season, alkalinity were high (41.1 meql^−1^) and salinity were also high (2.38 g l^−1^) during the dry season (Fig. 5). Nitrate concentrations were high (164.4 μg l^−1^) during the rainy season and low (30.1 μg l^−1^) during the dry season, while ammonia concentrations were high (80.4 μg l^−1^) during the post rainy season. Both soluble reactive phosphate (SRP) and total phosphate (TP) showed high values (0.66, 1.49 mg l^−1^) in the dry season and low values (0.26, 0.77 mg l^−1^) in the rainy season.

In Lake Chittu temperature was high during the post rainy and low during the dry season and dissolved oxygen showed high during the rainy season and low during the pre-rainy season. SRP and Tp showed high values during the dry and during the rainy seasons, respectively.

In Lake Shala temperature and pH showed higher values during the dry seasons while, dissolved oxygen and alkalinity showed higher values during the rainy season and low during the post rainy season. In the case of nutrients, ammonia and nitrate were lower during the post rainy season, where as SRP and TP showed higher values during the dry season and lower during the rainy season (Fig. 6).

### 3.3 Thermal regime

**Fig. 7:**
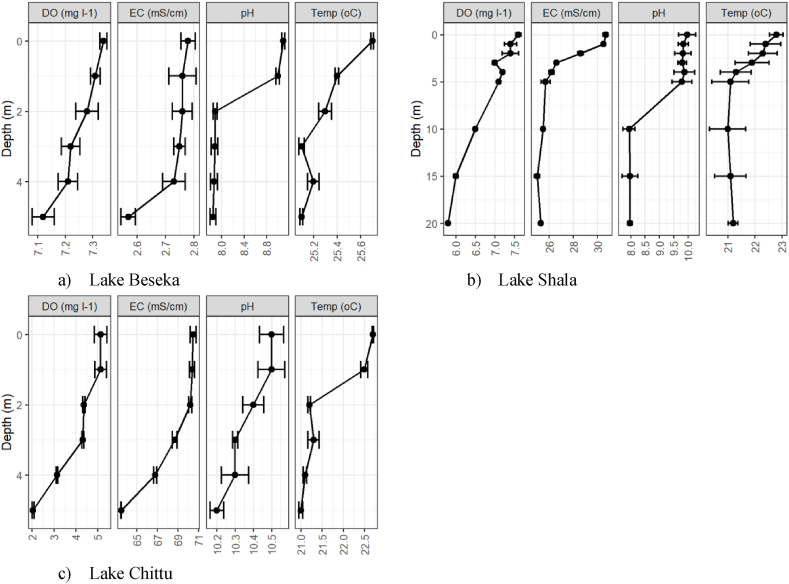
Dissolved oxygen (DO), Electrical conductivity (EC) and Water temperature depth profiles of the studied lakes.

Temperature and DO depth profiles in Lake Beseka exhibited an isothermal pattern with concentrations largely consistent and only slightly dropping below the fifth meter. Depth profiles of pH and conductivity showed some variations with depth, exhibiting decreasing trends (Fig. 7a). Surface water temperature and DO depth profiles in Lake Shala showed superficial thermal stratification with small temperature gradients. The mean temperature difference between surface water and 20 m depth was slightly higher (1.6 ± 0.25 °C). pH depth profiles recorded in Lake Shala at depths ranging from 0 to 20 m revealed variations with depth. The pH in the tropogenic zone (above 2 m depths), which was high (9.8) at the surface declined to 7.98 at 20 m depth. Conductivity depth profiles shows variation on the vertical section, first showing a decreasing trend and then increasing at 20 m depth (Fig. 7b). The vertical distribution of water temperature and DO in the water body of Lake Chittu demonstrated superficial stratification. Based on the measured data, the DO concentration in the surface layer (7.6 mg l^−1^) of Lake Chittu was decreased to (2.05 mg l^−1^) at the depth 5 m. Depth profiles of pH and conductivity of Lake Chittu for depths between 0 and 5 m indicated small decreasing variability with depth (Fig. 7c).

## 4 Discussion

### 4.1 Spatial variation of physicochemical parameters

In comparison to the other study lakes, Lake Beseka had the highest surface water temperature. This could be due to the lake's high volcano-tectonic forcing [44]. Dissolved oxygen is a critical component of an aquatic ecosystem, determining water quality and supporting aquatic life [45]. Dissolved oxygen concentrations below 5 mgl^−1^ have a negative impact on aquatic life. Among the studied lakes, Lakes Shala, Beseka, and Chittu had higher dissolved oxygen concentrations, whereas Lake Arenguade had the lowest dissolved oxygen concentration, which was close to 3.3 mgl^−1^. This could be due to the lake's altitude and the rapid decomposition of dead algae, as the lake is known to be productive [[46], [47], [48], [49]]. Other researchers also reported very low dissolved oxygen levels (less than 1 mg l^−1^) in other East African alkaline saline crater lakes [50].

Lakes Arenguade, Chittu, and Shala had higher pH, alkalinity, salinity, and electrical conductivity than Lake Beseka. This is due to the three lakes' high evaporation rates (high concentrations of dissolved minerals) and high concentrations of inorganic ions. Previous studies have reported similar results in these and other East African soda lakes [2,25,37,[51], [52], [53]]. The low pH, alkalinity, salinity and electrical conductivity of Lake Beseka can be explained by dilution due to rapid rise of lake water level [20,25].

Nitrate levels were lower in Lake Arenguade and Shala, which could be attributed to abundance of denitrifying bacteria, which lead to a potential loss of nitrate, aided by the favorable high tropical temperature [54,55]. This finding is consistent with other studies [27,51,52]. The relatively high level of nitrate concentration in Lake Beseka in comparison to other studied lakes is caused by continuous mixing of the lake and pollution from its catchment area. This may be due to the high amount of nitrate from sediments, decomposition and surrounding agricultural activities, entering the lake [46,47,56]. The higher ammonia, SRP and TP in Lake Chittu and Arenguade might be attributed to the predominance of phosphatic mineral-rich rocks, high evaporation rates exceeding precipitation in closed-basin carbonate-rich lakes [50,57,58]. Local community produces soda ash near the shore of Lake Chittu to feed their livestock as the salt licks in which animal excreta (cattle and flamingos) are expected to increase the ammonia content of the lake water. The huge number of grazing of livestock around the lakes may also contribute to increase in the nutrient levels through their droppings [59,60]. Oduor and Schagerl reported very high mammal population in Lake Nakuru National Park which contributes to the nitrogen levels through droppings in the lake [86]. This finding is consistent with some previous studies [14,25,52]. Similarly very high SRP concentrations up to 4 mg l^−1^ and TP concentrations up to 6 mg l^−1^ was recorded from saline-alkaline Lake Bogoria and Lake Nakuru of Kenya [61]. The low concentrations of ammonia, SRP and TP measured in Lake Beseka may be attributed to lake dilution due to its expansion [20,25]. The low concentration of HCO_3_ − + CO_3_^2−^ in Lake Beseka could be attributed to the lake's falling pH trend [62].

### 4.2 Temporal variation of physicochemical parameters

The physicochemical parameters of the four lakes, Lake Arenguade, Beseka, Chittu and Shala displayed considerable seasonal variation in relation to its dominant environmental conditions. Temperature is one of the important physical factors, which affects the physical properties, chemical reaction processes and biological activity in water, regulating the rate of photosynthesis in aquatic ecosystem. The higher water temperatures in lakes Beseka and Shala during the dry season could be attributed to high air temperatures [[63], [64], [65]]. The ambient temperature in the Lakes Beseka & Shala region is higher during the dry season (Jan–May). Higher DO concentrations during the rainy season in lakes Arenguade, Beseka and Chittu might be due to higher solubility of oxygen at lower temperature, low biological activity and mixing of surface water with atmospheric oxygen [46,[66], [67], [68]]. However, in Lake Shala the highest amount of DO was recorded during the dry season. This may be related to photosynthetic activities by aquatic plants and respiration rate of decomposers [46]. Thus, the current study conﬁrmed previous report on the seasonality of dissolved oxygen in Lake Shala [51].

Higher pH values recorded during the post rainy season in Lake Arenguade and in Lakes Beseka and Shala during the dry season might be attributed to the high evaporation rates and photosynthetic activities which decreases CO_2_ assimilation [46,69]. The conductivity values indicate that the higher conductivity was observed in dry season in Lakes Arenguade and Chittu, and in Lake Shala during the post rainy season which may be resulted from higher atmospheric temperatures resulting in higher evapotranspiration rates, high rate of decomposition and higher total ionic concentration [68,70]. This result is in agreement with the report of Solomon Wagaw from Lake Shala [51] and Zinabu G/Mariam [71] in some Ethiopian rift valley lakes, but contrasts with the report by Taddese Ogato [23].

The high level of salinity in the dry season in Lakes Arenguade, Beseka and Shala may be attributed to increased evaporative concentrations of salts due to higher temperatures [72]. This agrees with Solomon Wagaw who reported high salinity level during the dry season in Lake Shala [51]. Alkalinity levels were high during the post-rainy season in Lakes Beseka and Chittu, and during the dry season in Lake Shala. This may likely be caused by high rate of decomposition, which releases CO_2_ and results in the addition of carbonate and bicarbonate ions, increasing alkalinity [73].

Ammonia concentrations were high in Lakes Arenguade and Beseka after the rainy season and in Lake Chittu during the dry season. There is an agricultural activity surrounding the catchment of Lake Arenguade and Beseka in which nutrients directly released into the lake in the form of runoff and soil erosion during the rainy season and accumulates as the water begins to evaporate during the post rainy and dry seasons [74]. During the dry season, the high concentration of nitrate in Lake Arenguade and Shala might be due to runoff from the surrounding area, rich in wild and domestic animal feces, causing accelerated eutrophication. Large number of flamingos, other aquatic birds and livestock are usually observed on the shores of these two lakes [61,75].

Phosphate plays a major role in primary productivity in an aquatic ecosystem as it promotes growth for organisms and limits the phytoplankton production. The high concentration of both SRP and TP in Lakes Beseka, Chittu and Shala during the dry season can be attributed to increased temperatures which impact the seasonal retention and release of phosphorus and decay and subsequent mineralization of dead organic matter [68]. In addition, the waves disturb the sediments thereby bringing more phosphate from the sediments [46,69,76].

The higher concentration of silicate recorded during the rainy season in Lakes Arenguade and Beseka, during the post rainy season in Lake Shala might be due to the enhanced dissolution of solid silicates [27]. High consumption of silicate by diatoms during the dry season might have contributed to the less availability of silicate concentration during dry seasons [77].

### 4.3 Thermal regime

The water temperature in Lake Beseka's surface layer was impacted by external climatic factors such as wind and the depth profile revealed an isothermal pattern [78]. Similarly, DO depth profiles show relatively uniform trend which might be due to continuous mixing of the lake. Surface water temperature and DO depth profiles in Lake Shala showed superficial thermal stratification which might be due to the occurrence of wind-induced turbulent mixing favored by absence of crater walls and vegetation cover from wind action. This result is in accordance with the results by Tadesse Ogato [79]. The pH change in the water body was linked to the photosynthesis of aquatic organisms because the enough light at the surface water aided vigorous photosynthesis, which increased the pH. In the bottom, the paucity of light reduced photosynthetic activity, which lowers pH [78]. The vertical distribution of water temperature and DO in Lake Chittu's water body revealed superficial stratification. The downward trend in DO could be attributed to a dense population of Arthrospira fusiformis protecting the light entrance, as well as a poor rate of photosynthesis and organic matter decomposition [52].

### 4.4 Long term selected physicochemical trends

**Fig. 8:**
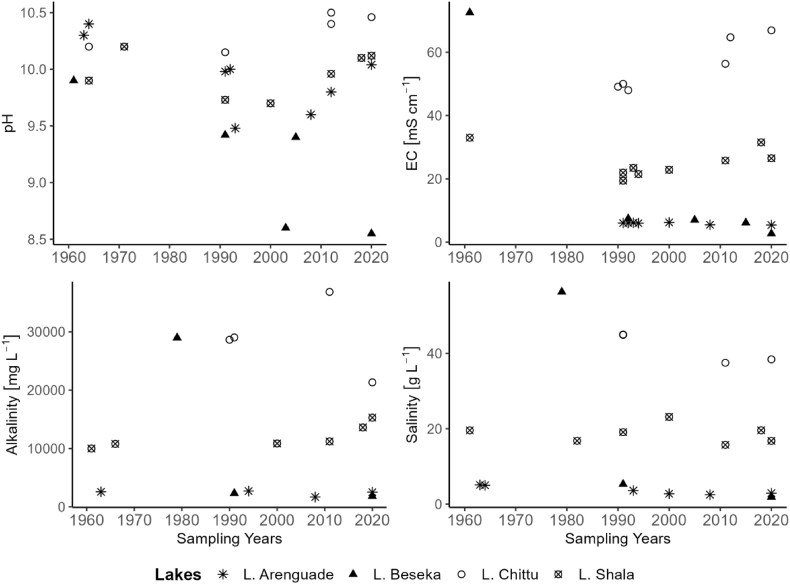
Trends in physicochemical parameters of studied lakes since 1960–2020.

A historical summary of previous studies on selected physicochemical variables of the studied lakes across years for the long-term data from 1961 to 2020 showed variations (Fig. 8). Comparing the current mean pH to studies conducted in Lake Arenguadé between 1961 and 2012, pH frequently declined from 10.4 [48] to 9.48 in 1990s to 9.6 in 2008 [14] but it showed small increase (10.04) in the present study. Conductivity values for the period 1991 to 2020 also declined regularly from 6.05 mS/cm in the 1990s to 5.5 mS/cm in 2008 and 5.42 mS/cm in the present study. The alkalinity of Lake Arenguade decreased from 51.4 meql^−1^ in the 1960s to 33.5 meql^−1^ in 2008, but the alkalinity values in this study are comparable to the average value of the 1960s (50.2 meql^−1^). The salinity of Lake Arenguade, has dropped from 5.1 g l^−1^ of the 1960s [48]to 2.5 g l^−1^ of 2008 [14], showing slight increase (2.91 g l^−1^) in the present study. In general, long-term trends of physicochemical parameters of Lake Arenguade showed a decreasing trend from 1961 to 2008 but are now slightly increasing. Lake Arenguade is becoming more diluted as a result of frequent high-velocity explosions conducted for seismological research that have altered the lake's water chemistry and plankton diversity [14]. Recurrent drought might be the reason for the current minor increase in physico-chemical features, which enhances evaporation leading to rise in physicochemical parameters [9,80].

Lake Beseka's pH, conductivity, alkalinity, and salinity, all decreased significantly when compared to 1960s. The mean pH dropped from 9.9 in the 1960s to 9.4 in the 1990s to 8.6 in 2003 and 8.55 in the current study. Similarly, mean conductivity values fell from 72.5 mS/cm [37] in the 1960s to 7.441 mS/cm [81] in the 1990s and to 2.72 mS/cm in the present study. The mean alkalinity values have been decreasing from 580 meql^−1^ [27] in 1970s to 67.6 meql^−1^ in 2020. The lake's salinity has also decreased dramatically, from 56.3 g l^−1^ in the 1970s to 5.3 g l^−1^ in the 1990s and currently to 1.88 g l^−1^. The increasing water level of the lake may be a contributing factor to the declining trend of the physicochemical parameters of Lake Beseka [44]. Similar findings were made on Lake Baringo in Kenya, where changes in lake water levels had an impact on the physicochemical characteristics of the lake [78].

When compared to previous studies conducted in Lake Chittu from 1971 to 2012 and Lake Shala from 1961 to 2018, the mean pH values showed a slightly increasing trend during the current study, but remained within the ranges found in the previous studies. Mean conductivity values in the present study of Lake Chittu were, generally, higher in contrast to records from the 1990s, which was 50 mS/cm [82] and 66.9 mS/cm in the present study. Lake Chittu's alkalinity decreased t from 573 meql^−1^ [83]in 1990s to 426.8 meql^−1^ in 2020, but Lake Shala's alkalinity increased from 200 meql^−1^ [84] in 1960s to 305.7 meql^−1^ in 2020. In the 1990s, Lake Chittu's mean salinity result was higher (45 g l^−1^) [82] than it was in the 2010s (37.5 g l^−1^) [36], but it is currently 38.41 g l^−1^. The results from the 1980s (16.8 g l^−1^) [53] are comparable to the current mean salinity readings (16.78 g l^−1^) for Lake Shala, while it was slightly higher in the year 2000 (23.15 g l^−1^) [85]. Very small rises in physicochemical parameters of Lake Chittu and Shala could be the result of higher evaporative concentrations due to recurrent drought and anthropogenic activities in the catchment [6,9].

## 5 Conclusions

The physico-chemical parameters investigated in the current study showed variability depending on location, season and trends across years. All physicochemical variables showed significant differences between the sampling seasons (p < 0.05), except salinity in Lake Shala. The concentrations of physicochemical parameters were, generally, high during the dry seasons in the studied lakes due to the low incidence of rainfall caused by climate change, resulting in higher evapotranspiration rates as they are characterized by a long dry season. Lakes Arenguade and Beseka showed a considerable decrease in conductivity, alkalinity and salinity compared to data from the 1960s and 1990s but are now showed slightly increasing trend in Lake Arenguade which might be due to high evaporation rate. Longterm physicochemical trend in Lakes Chittu and Shala showed very small rise in physico-chemical parameters which could be a result of higher evaporative concentrations due to climate change and catchment activities. The general condition of the four lakes shows that there is little or no change over time compared to historical data. This can be attributed to the high carbonate-bicarbonate buffer zone that resists change in pH that governs most of the physicochemical parameters. This study has a limitations that it was conducted over a short-term period of time to see a long-term change. Robust sample size and long-term study is required to visualize clear trend of change in physicochemical alterations of the rift system. However, the data generated from this study can be used as baseline for further future studies as source of monitoring and establishment of management strategies and devising mitigation measures for water and biodiversity conservation.

## Author contribution statement

Hana Melese Bekele: Conceived and designed the experiments; Performed the experiments; Analyzed and interpreted the data; Wrote the paper. Habte Jebessa Debella: Conceived and designed the experiments; Contributed reagents, materials, analysis tools; Wrote the paper.

## Data availability statement

Data will be made available on request.

## Funding

This research was funded by 10.13039/501100007941Addis Ababa University, School of Graduate Studies Program and Thematic Research Projects.

## Declaration of competing interest

The authors declare that they have no known competing financial interests or personal relationships that could have appeared to influence the work reported in this paper.
